# Supplementary material for: Diffusion in translucent media
Source: Nat Commun. 2018 May 10;9:1862. doi: 10.1038/s41467-018-04242-4 (PMC5945602; doi:10.1038/s41467-018-04242-4)
Supplement: Supplementary file 1 — Supplementary Information [file 41467_2018_4242_MOESM1_ESM.pdf]

Supplementary Information for "*Diffusion in translucent media*"

Shi et al.

Supplementary Information for "*Diffusion in translucent media*"

Zhou Shi[1,2] and Azriel Z. Genack[1]

[1]*Department of Physics, Queens College and Graduate Center of the City University of New York, Flushing, New York 11367, USA*

[2]*Chiral Photonics Inc. 26 Chapin Road, Pine Brook, NJ 07058*

Supplementary Note 1 - Spatial parameters in the diffusion model

The flow of incoherent wave energy in an unbounded non-dissipative medium is described by the diffusion equation (*1-4*),

$$\frac{\partial u(\mathbf{r},t)}{\partial t} = -D\nabla^2 u(\mathbf{r},t) = Q(\mathbf{r},t) \qquad (1)$$

Here $u(\mathbf{r}, \mathrm{t})$ is the energy density, $D = v\ell/d$ is the diffusion coefficient, v is the transport velocity, ℓ is the transport mean free path, d is the dimensionality, and $Q(\mathbf{r}, t)$ is a source function of incoherent waves. The diffusion equation only describes the evolution of energy density of fully randomized waves, however, the diffusion model can provide the evolution of energy density created by a coherent incident beam incident within a bounded sample by phenomenologically incorporating interactions at the interface (*1-4*). A coherent incident beam is replaced with a delta function source of incoherent radiation at a depth z_p into the sample. For an incident beam with angle of refraction within the medium θ, the penetration depth is $z_{p=}$ $z_{p0}\cos\theta$, where z_{p0} is the penetration depth for a normally incident beam (*4*). The boundary may be eliminated by solving the diffusion equation in an unbounded medium in which the linearly decaying intensity near the boundaries on either side of the source extrapolates to zero at a distance z_b beyond the sample. This is illustrated in Supplementary Figure 1.

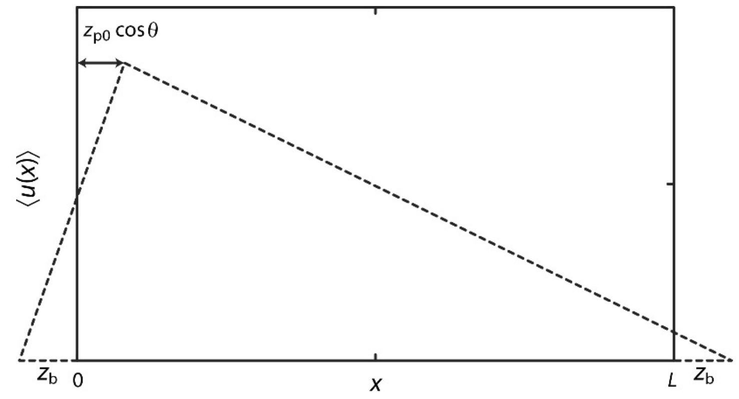

Supplementary Figure 1| Illustration of the diffusion model within a bounded random medium. Interactions at the boundaries are incoporated using phenomenological lengths. An incoherent beam with refracted angle θ is replaced by an isotropic source at a depth $z_{p0}\cos\theta$ into the sample from which the energy density diffuses freely. The energy density near the boundaries extrapolates to zero at a distance z_b beyond the sample's open surfaces.

In this model, the reflected flux, for unit incident flux in channel a, $\langle R_a\rangle$ is the flow to the left from the source at $z_{p0}\cos\theta$, while the transmitted flux, $\langle T_a\rangle$, is the flow to the right from the internal source. These fluxes are given by $-D\frac{du(x)}{dx}$, according to Fick' first law. The gradients of the energy density are proportional to the inverse of the distance from z_p to the points beyond the sample at which the energy density extrapolates to zero, so that $\frac{\langle T_a\rangle}{\langle R_a\rangle}=\frac{z_{p0}\cos\theta+z_b}{L+z_b-z_p}$. Together with the condition for conservation of energy, $\langle R_a\rangle+\langle T_a\rangle=1$, this gives (*4*),

$$\langle T_a\rangle=\frac{z_{p0}\cos\theta+z_b}{L+2z_b} \tag{2}$$

This expression is verified in measurements of optical transmission vs. L, in which the incident angle is varied in samples which are and are not index matched to their surroundings (*4*).

We find an expression for the transmission averaged over random configurations and over all incident channels, $\langle\langle T_a\rangle\rangle_a=\langle T\rangle/N=u(L)v_+=W(L)$, by considering the energy density, $u(x)$, associate with the transmittance $\langle T\rangle$, as shown in Supplementary Figure 2. Here, v_+ is the average magnitude of the longitudinal speed of the wave in the medium. The simulations presented in Fig. 2c of the main text of the normalized energy density within a random medium

shows that $\langle W(x) \rangle$ falls linearly within samples shorter than the localization length from $W(0)=2-\langle T \rangle /N$ at the incident surface to $W(L)=\langle T \rangle /N$ at the output of the sample.

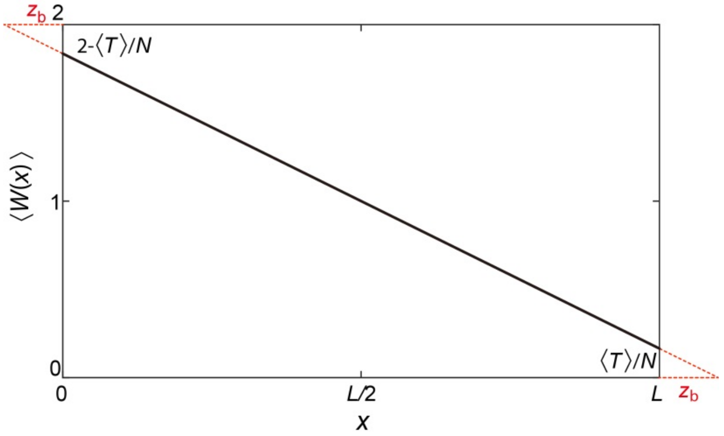

Supplementary Figure 2| Linear falloff of W(x) within a scattering medium and its extrapolation beyond the sample. W(x) extrapolates to 0 at L+zb and to 2 at -zb.

Since the gradient of the average energy density is, $\frac{du(x)}{dx} = \frac{dW(x)}{v_+ dx} = -\frac{\langle T \rangle / N}{z_b v_+}$, the flux at the output $x = L$ is

$$\frac{\langle T \rangle}{N} = -\frac{v\ell}{2}\frac{du(x)}{dx} = \frac{v\ell \langle T \rangle / N}{2v_+ z_b}. \tag{3}$$

This gives

$$z_b = v\ell / 2v_+. \tag{4}$$

Since $\langle W(x) \rangle$ extrapolates to zero at $x = L + z_b$ and to 2 at $x = -z_b$, $\frac{du(x)}{dx}$ can also be expressed as

$$\frac{du(x)}{dx} = -\frac{2}{(L + 2z_b)v_+}. \tag{5}$$

The flux at the output is thus given by

$$\frac{\langle T\rangle}{N}=\frac{(v/v_+)\ell}{L+2z_\mathrm{b}}. \quad (6)$$

This may be compared to the average of Supplementary Equation 2 over all incident channels,

$$\langle T_\mathrm{a}\rangle_\mathrm{a}=\frac{\langle T\rangle}{N}=\frac{\overline{z}_\mathrm{p}+z_\mathrm{b}}{L+2z_\mathrm{b}}. \quad (7)$$

where $\overline{z}_\mathrm{p}$ is the average of z_p over all incident channels. This gives $\overline{z}_\mathrm{p}+z_\mathrm{b}=(v/v_+)\ell$ and

$$\overline{z}_\mathrm{p}=z_\mathrm{b}, \quad (8)$$

in the case that the sample is index matched and there is no reflection at the sample's longitudinal boundaries.

Supplementary Note 2 - Universality of structure of x_n for waves in random media

The x_n, which are related to the transmission eigenvalues via $\tau_\mathrm{n}=1/\cosh^2 x_\mathrm{n}$ (*5*, *6*), are seen in Fig. 3a of the main text to be equally spaced for $n < N/2$ in both translucent and diffusive samples. For opaque samples, $L >> \ell$, the spacing between the x_n for $n < N/2$ is predicted to be the inverse of the bare conductance, $\Delta x = 1/g_0 \sim L/N\ell$, in which the renormalization of the conductance by coherent backscattering and boundary effects are not included (*5*,*6*). Measurements in samples with L not much larger than ℓ, show that $\Delta x = (L+2z_\mathrm{b})/\eta N\ell$ with $\eta \sim 1$ (*7*). This suggests that when the effect of surface reflectivity is taken into account, the bare conductance is given by $g_0=\eta N\ell/(L+2z_\mathrm{b})$. Since Supplementary Equation (4) and (6) yield $\langle T\rangle = Nz_\mathrm{b}/(L+2z_\mathrm{b})$, this gives $\eta = z_\mathrm{b}/\ell = v/2v_+$.

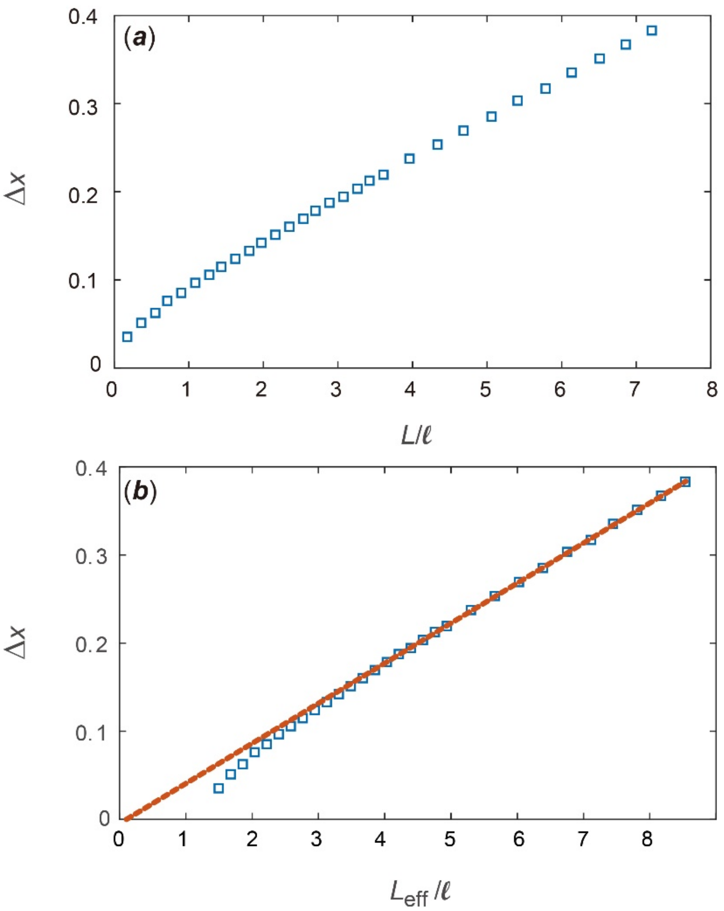

Supplementary Figure 3| Scaling of Δx vs L/ℓ and L_{eff}/ℓ. (a) The average spacing between adjacent x_n rises rapidly at first before increasing linearly in the diffusive regime. **(b)** The red dashed line in Fig. 3b is a linear fit to the data for $L_{eff}/\ell > 3$ and is seen to intercept $\Delta x = 0$ approximately at $L_{eff} = 0$ or equivalently $L = -2z_b$.

The red dashed line in Fig. 3b is a linear fit to the data for $L_{eff}/\ell > 3$ and is seen to intercept $\Delta x = 0$ approximately at $L_{eff} = 0$ or equivalently $L = -2z_b$.

For translucent samples, Δx is not proportional to L, but varies linearly with L for L > ℓ, as seen in Supplementary Fig. 3a. Supplementary Figure 3b shows that for L>ℓ, Δx is proportional to (L+2zb)/ℓ and extrapolates to zero at L = -2zb. Thus (L+2zb) is an effective length Leff, that is determined from the scaling of the xn.

The probability distribution of the x, $\rho(x,L)$, for various lengths normalized by L/ℓ or by L_{eff}/ℓ is shown in Supplementary Figs. 4a,b. For the two diffusive samples considered, $\rho(x,L)$

collapses to a single curve when normalized by L_{eff}/ℓ, suggesting the universal distribution of $\rho(x,L)$ for opaque diffusive samples, $L >> \ell$. The universal distribution breaks down, however, for translucent samples, $L << \ell$. The distribution $\rho(x,L)$ of x normalized by Δx, is universal for both translucent and diffusive samples, as seen in Supplementary Fig. 4c.

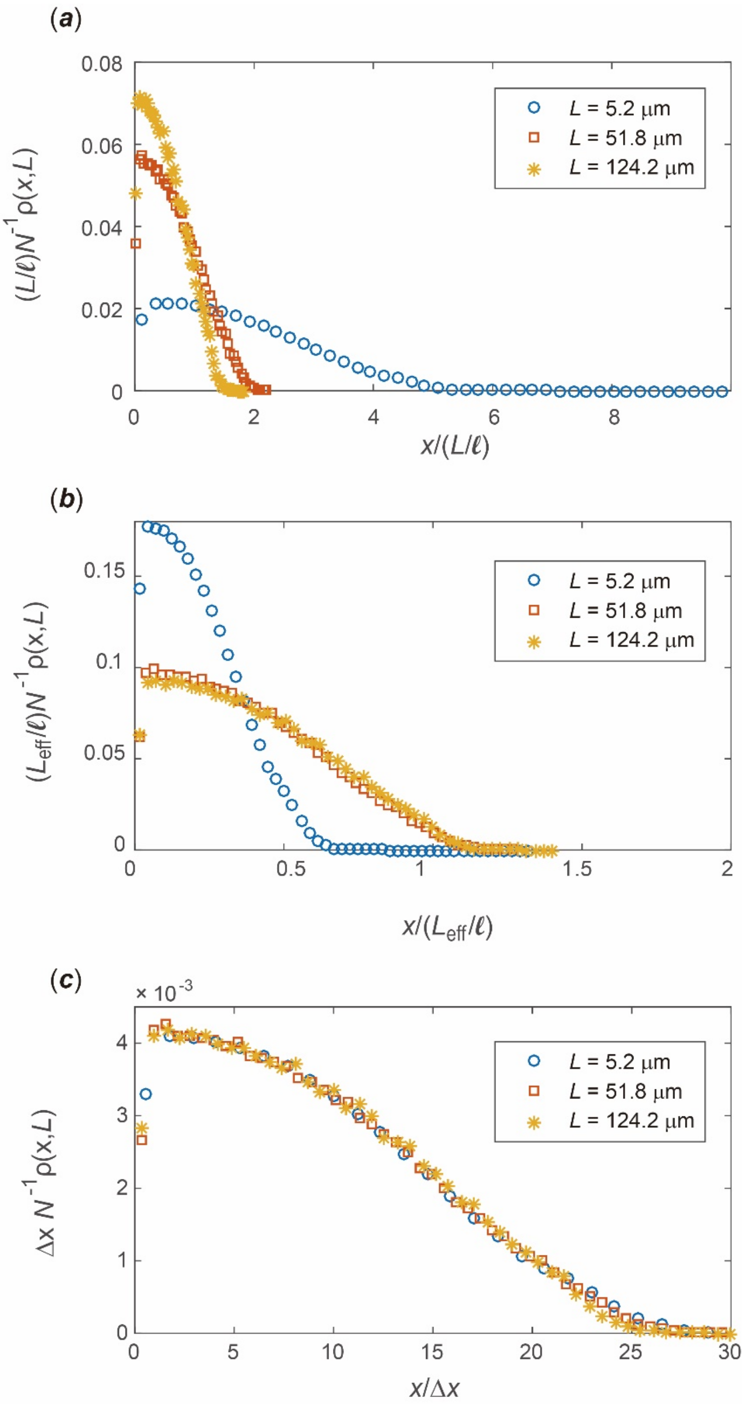

Supplementary Figure 4| Universal structure of the density of x_n. (a-c) Probability distribution of $\rho(x,L)$ plotted vs. $x/(L/\ell)$, $x/((L+2z_b)/\ell)$ and $x/\Delta x$, respectively.

Supplementary Note 3 - Expression for $S_\tau(x/L)$ for diffusive and translucent media

For an eigenchannel of a diffusing system with eigenvalue τ, the expression for $S_\tau(x/L)$ is found in simulations to be

$$S_\tau(x/L) = 2\frac{\cosh^2(h(x/L)(1-x/L)(L/\xi'))}{\cosh^2(h(x/L)L/\xi')} - \tau, \tag{9}$$

where $h(x/L)$ is an empirical function and $\tau = 1/\cosh^2(L/\xi')$ (8). $h(x/L)$ is found by comparing Supplementary Eq. (9) with the results of simulations for a specific value of τ. With the function $h(x/L)$ obtained in this way, excellent agreement is found between simulation and Supplementary Eq. (9) in diffusive samples for all values of τ. The need for the empirical function $h(x/L)$ for diffusive samples is seen in the comparisons in Supplementary Fig. 4 for a sample with L = 124.2 µm, of the simulation results, Supplementary Eq. (9), and the expression in Supplementary Eq. (9), but without the empirical function $h(x/L)$. This demonstrates the need for the empirical function $h(x/L)$ in diffusive samples. In contrast, as shown in Fig. 4c of the main text, good agreement is found between simulations and the expression in Supplementary Eq. (9) without the empirical function $h(x/L)$ in translucent samples. This is illustrated in Supplementary Fig. 5 for τ= 0.7 in a sample with L = 5.2 µm.

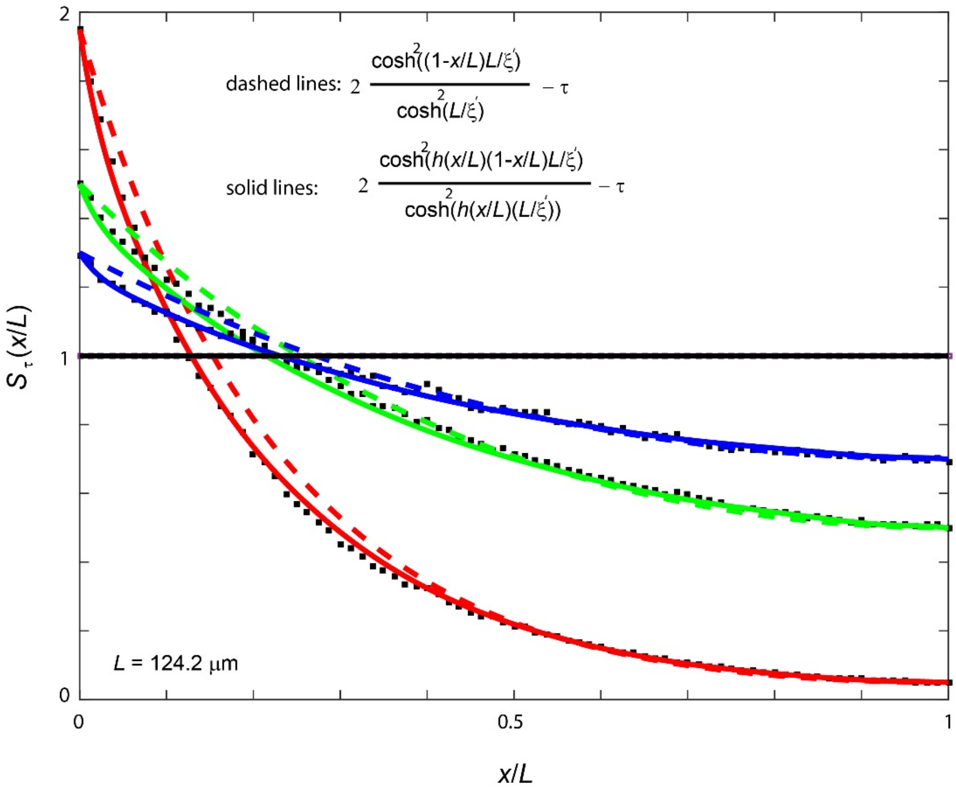

Supplementary Figure 5| Comparison of the simulations with the expression for $S_\tau(x/L)$ for various values of τ for a diffusing sample with L = 124.2 μm. The symbols are the points obtained in simulations for τ = 1, 0.7, 0.5 and 0.01, as indicted by the value of $S_\tau(1)=\tau$.

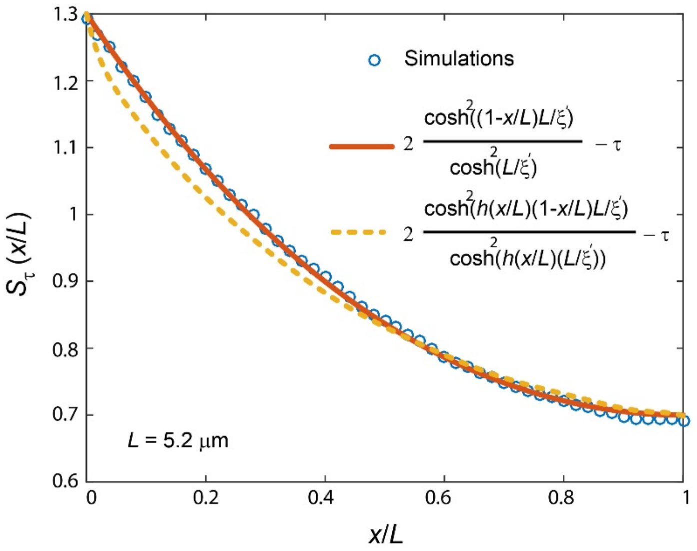

Supplementary Figure 6| $S_\tau(x/L)$ in a translucent sample of L = 5.2 μm. Comparison of the simulation results with expression for $S_\tau(x/L)$ for $\tau = 0.7$ in a sample with and without the empirical factor $h(x/L)$.

Supplementary Note 4 - Scaling of the eigenchannel delay time of fully transmitting t_1 and the delay time t_D and the scaling of τ_n

The transmission delay time t_D is equal to the sum of the single channel delay time weighted by the intensity, $t_D = \sum_{a,b=1}^{N} |t_{ba}|^2 \frac{d\varphi_{ba}}{d\omega} / \sum_{a,b}^{N} |t_{ba}|^2$, where $\frac{d\varphi_{ba}}{d\omega}$ is the derivative of the phase of t_{ba} with respect to the angular frequency ω (*9-11*). The delay time is also proportional to the density of states of the sample and to the sum of the energy stored within the medium for each of the eigenchannels (*8,12-15*). Alternatively, t_D may be obtained from the sum of the differences in the derivative with angular frequency of the composite phase of the transmission eigenchannel on the outgoing and incoming surfaces of the sample summed over all transmission eigenchannels (*15*). The eigenchannel dwell time, t_n, is proportional to the contribution of the eigenchannel to the density of the states, and the energy stored within the sample in a transmission eigenchannel.

The delay time t_D can also be expressed in terms of the t_n and τ_n, $t_D = \sum_1^N \tau_n t_n / \sum_1^N \tau_n$. The delay time of the eigenchannel is given by integrating the energy density distribution of the eigenchannels over the sample, $t_n \propto \int_0^L W_n(x)dx$. The same results are obtained when the eigenchannel delay time is obtained from the spectral derivative of the composite phase associated with the transmission eigenchannel, $t_n = \frac{d\theta_n}{d\omega}$, where $\frac{d\theta_n}{d\omega} = \frac{1}{i}(u_n^* \frac{du_n}{d\omega} - v_n^* \frac{dv_n}{d\omega})$ (*8*). Since both t_n and τ_n fall rapidly once τ_n <1/e, t_D is dominated by the g open transmission eigenchannels. The scaling of various τ_n and t_n/t_B are shown in Supplementary Figure 7.

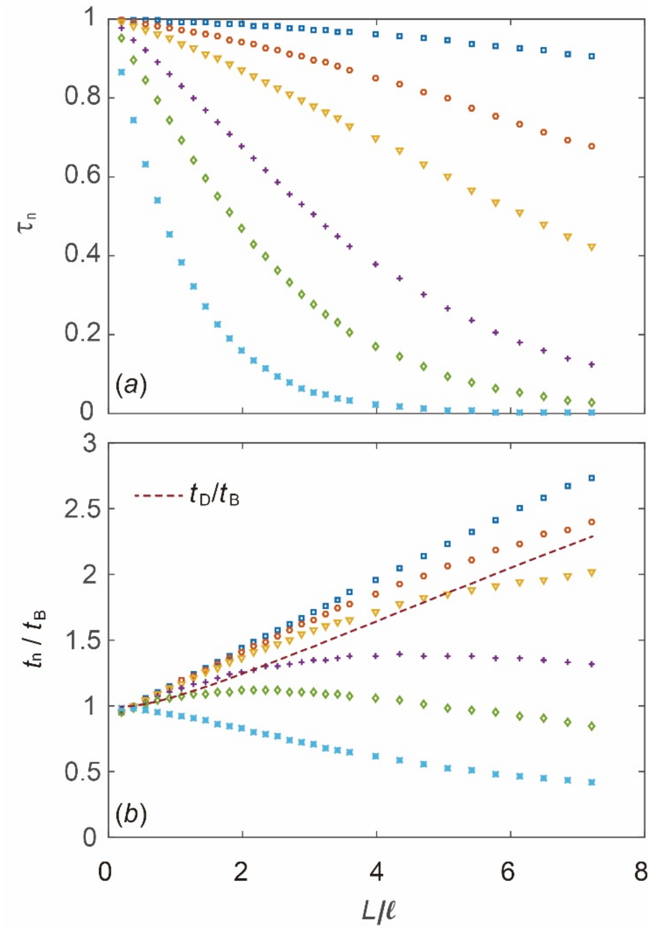

Supplementary Figure 7| Scaling of transmission eigenchannels and dwell times. Scaling of τ_n and t_n/t_B for n = 1, 2, 3, 5, 7 and 10 (from top to bottom) vs. L/ℓ.

Computing the integral over the sample length L of the energy density profile for the fully transmitting eigenchannel, $W_1(x)=1+F_1(x/L)=1+A[4(x/L)(1-x/L)]$, to give t_1 yields $t_1 \propto L+\frac{2}{3}AL$. For $L < \xi$, the value of A can be found by considering the return probability at the center of the sample, $A=\frac{Lv_+}{4D}$ (*8*). For 2D samples, $D = v\ell/2$ and therefore $A = v_+L/2v\ell$. This gives

$$t_1 \propto L+\frac{v_+}{3v\ell}L^2 \tag{10}$$

From Supplementary Eq. (10), it is clear that only when L is substantially greater than the transport mean free path is the delay time of the highest and other high-transmission eigenchannels, t_1 and t_τ, scale substantially faster than linearly.

Supplementary Note 5 - Scaling of delay time t_D with the effective length L_{eff}

We have seen in measurements, simulations and calculations that transmission scales inversely with the effective length, $L_{eff} = L+2z_{b}$., We now consider the role of L_{eff} in the scaling of dynamics. Supplementary Figure 8 shows that t_D is proportional to L_{eff}^2 once $L_{eff} > 4\ell$. Thus the dynamics of diffusive transport depends on z_b even though z_b does not enter explicitly in the expressions for τ_n or $W_n(x)$.

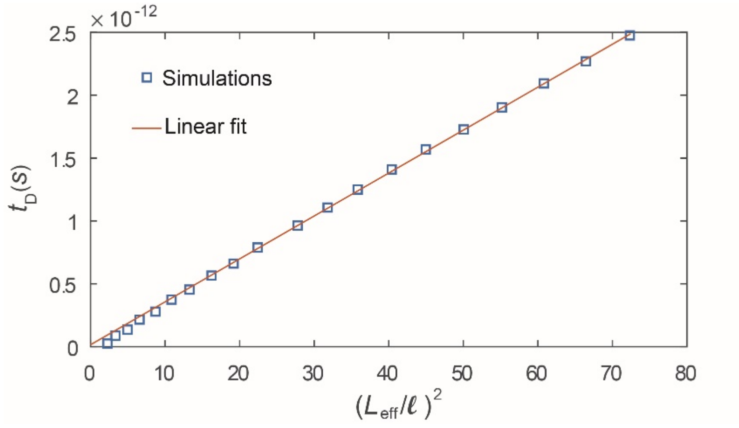

Supplementary Figure 8| Scaling of t_D. t_D reflects the scaling of all the t_n and τ_n. It scales quadratically with L_{eff} for $L_{eff}/\ell > 4$.

The quadratic scaling for larger lengths extrapolates to $t_D = 0$ at $L_{eff} = 0$. The linear fit is done for $(L_{eff}/\ell)^2 > 10$.

Supplementary References

1. Van Rossum, M. C. W. & Nieuwenhuizen, Th. M. Multiple scattering of classical waves: microscopy, mesoscopy, and diffusion, *Rev. Mod. Phys.* **71,** 313-371 (1999).
2. Akkermans, E. & Montambaux, G. *Mesoscopic physics of electrons and photons* (Cambridge University Press, 2007).
3. Zhu, J. Pine, D. J. & Weitz, D. A. Internal reflection of diffusive light in random media. *Phys. Rev. A* **44**, 3948-3959 (1991).

4. Li, J. H., Lisyansky, A. A., Cheung, T. D., Livdan, D. & Genack, A. Z. Transmission and surface intensity profiles in random media. *Europhys. Lett.* **22**, 675-680 (1993).
5. Dorokhov, O. N. On the coexistence of localized and extended electronic states in the metallic phase. *Solid State Commun.* **51**, 381–384 (1984).
6. Beenakker, C. W. J. Random-matrix theory of quantum transport, *Rev. Mod. Phys*. **69**, 731-808 (1997).
7. Shi, Z. & Genack, A. Z. Transmission eigenvalues and the bare conductance in the crossover to Anderson localization. *Phys. Rev. Lett.* **108**, 043901 (2012).
8. Davy, M., Shi, Z., Park, J., Tian, C. & Genack, A. Z. Universal structure of transmission eigenchannels inside opaque media. *Nature Commun.* **6**, 6893 (2015).
9. Genack, A. Z., Sebbah, P., Stoytchev, M., & van Tiggelen, B. A., Statistics of Wave Dynamics in Random Media, *Phys. Rev. Lett.* **82**, 715-718 (1999).
10. Wigner. E., Lower Limit for the Energy Derivative of the Scattering Phase Shift, *Phys. Rev.* **98**, 145-147 (1955).
11. Smith, F. T. Lifetime Matrix in Collision Theory, *Phys. Rev.* **118**, 349-356 (1960).
12. Avishai, Y. & Band, Y. One-dimensional density of states and the phase of the transmission amplitude. *Phys. Rev. B* **32**, 2674-2676 (1985).
13. Iannacone, G. General relation between density of states and dwell times in mesoscopic systems. *Phys. Rev. B* **51**, 4727–4729 (1995).
14. Brandbyge, M. & Tsukada, M. Local density of states from transmission amplitudes in multichannel systems, *Phys. Rev. B* **57**, R15088 (1998).

15. Davy, M, Shi, Z., Wang, J., Cheng, X. & Genack, A. Z. Transmission eigenchannels and the densities of states of random media. *Phys. Rev. Lett.* **114**, 033901 (2015).
